# Understanding the Impact of Biodegradable Microplastics on Living Organisms Entering the Food Chain: A Review

**DOI:** 10.3390/polym15183680

**Published:** 2023-09-06

**Authors:** Konstantin V. Malafeev, Annalisa Apicella, Loredana Incarnato, Paola Scarfato

**Affiliations:** Department of Industrial Engineering, University of Salerno, Via Giovanni Paolo II n. 132, 84084 Fisciano, SA, Italy; kmalafeev@unisa.it (K.V.M.); lincarnato@unisa.it (L.I.); pscarfato@unisa.it (P.S.)

**Keywords:** biodegradable microplastics, plants, fish, ecotoxicity, pollution, plant biomass

## Abstract

Microplastics (MPs) pollution has emerged as one of the world’s most serious environmental issues, with harmful consequences for ecosystems and human health. One proposed solution to their accumulation in the environment is the replacement of nondegradable plastics with biodegradable ones. However, due to the lack of true biodegradability in some ecosystems, they also give rise to biodegradable microplastics (BioMPs) that negatively impact different ecosystems and living organisms. This review summarizes the current literature on the impact of BioMPs on some organisms—higher plants and fish—relevant to the food chain. Concerning the higher plants, the adverse effects of BioMPs on seed germination, plant biomass growth, penetration of nutrients through roots, oxidative stress, and changes in soil properties, all leading to reduced agricultural yield, have been critically discussed. Concerning fish, it emerged that BioMPs are more likely to be ingested than nonbiodegradable ones and accumulate in the animal’s body, leading to impaired skeletal development, oxidative stress, and behavioral changes. Therefore, based on the reviewed pioneering literature, biodegradable plastics seem to be a new threat to environmental health rather than an effective solution to counteract MP pollution, even if serious knowledge gaps in this field highlight the need for additional rigorous investigations to understand the potential risks associated to BioMPs.

## 1. Introduction

In 2015, the United Nations General Assembly adopted the “2030 Agenda for Sustainable Development”, a comprehensive plan of action having at its core 17 sustainable development goals (SDGs) to transform our world. According to the targets associated with them, to ensure a satisfactory transition towards sustainable consumption and production patterns, it is necessary to significantly reduce the amount of waste through the adoption of measures to prevent its generation and to reduce, recycle, and reuse products or components [1]. This task is directly related to solving the problem of polluting the planet with plastic waste. Polymers have firmly taken their place in all areas of our lives during the 20th century. Gradually, when plastic products fail, waste plastic accumulates in landfills and beyond. Eventually, nondegradable plastics begin to decompose under the influence of the environment (humidity, UV radiation, etc.) and release microplastics. Microplastics are plastic particles ranging in size from 1 µm to 5 mm [2]. Recently, the number of studies on the impact of microplastics on the environment has been growing; the presence of microplastics has been recorded in Antarctica [3], as well as in the human placenta [4]. This indicates that microplastics have firmly entered our lives and will affect the environment [5]. Microplastics carry many threats. The main ones are: (i) entering living organisms with food and accumulation within them; (ii) formation of bacterial biofilms on their surface and the spread of bacteria to regions unusual for them; and (iii) changes in soil parameters, which will reduce land productivity [2,6,7]. All this entails the destruction of the planet’s ecosystems. One way to clean up pre-existing microplastics is by enzymatic degradation [8]. However, it is also necessary to reduce the use of nondegradable plastics.

**Figure 1:**
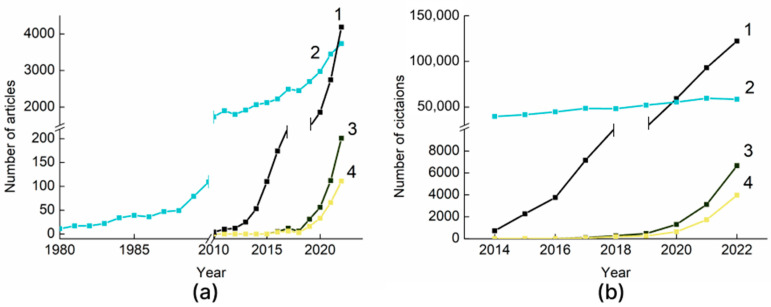
Statistics from the Scopus database: (**a**) number of articles and (**b**) number of citations database on request: 1—microplastics; 2—biodegradable polymers; 3—biodegradable microplastics; 4—biodegradable microplastics pollution.

In this perspective, research is underway to replace conventional nondegradable plastics with biodegradable ones (PLA, PBAT, PBS, etc.) [9]. Studies on “biodegradable polymers” started in the 1980s and their number is continuously increasing (Figure 1a), driven by the belief that biodegradable plastics are a panacea for plastic pollution. This idea has also prompted huge market investments in this area [10]. According to the latest market data compiled by European Bioplastics, global bioplastics production capacities are set to grow from around 2.23 million tons in 2022 to approximately 6.3 million tons in 2027 [11]. In 2019 the market for biodegradable plastic packaging was valued at USD 4.65 million, and, by the end of 2025, it is expected to grow at a CAGR of 17.04% reaching a market value of up to 12.06 million [12].

However, even if theoretically most biodegradable polymers decompose into environmentally acceptable products, such as water, carbon dioxide, and biomass, this is only achievable under physiological conditions or controlled industrial-composting conditions [13]. In real life, when biodegradable polymers end up in natural environments, their biodegradation is affected by several chemical–physical (oxygen availability, light, pH, temperature, humidity) and biological (microorganism and enzyme type and concentration) factors. Therefore, the biodegradation rate and the degradation profile can also vary significantly to the point where they can long persist in the environment. This leads to the weathering of plasticizers, as well as the formation of BioMPs [14]. The field of study of biodegradable microplastics is quite young; according to the Scopus database, the first studies on this topic appeared in 2016. Since then, the number of articles on “biodegradable microplastics” has grown exponentially, from 5 articles in 2016 to 201 articles in 2022. Also, the first articles on “biodegradable microplastics pollution” appeared in 2016. Their number shows the same trend also (Figure 1a). This trend will continue in the coming years, with the increase of biodegradable polymers in our lives and the obtaining of new data on the impact of biodegradable microplastics on the environment. The problem of BioMPs can become as urgent as the problem of MPs. Figure 1a shows an exponential increase in the number of publications for the query “microplastics”. By the number of articles per year, microplastics are already ahead of biodegradable polymers. Figure 1b also points out the relevance of the topic of microplastics, providing citation data for articles related to “microplastics” and “biomicroplastics”. It can be seen that the topic of “biodegradable polymers” is already well-established, and, annually, the number of citations of the 2000 most-cited articles is about 50,000. At the same time, the citation of the 2000 most-cited articles related to microplastics continues to grow, and, in 2022, it reached 150,000. Articles related to BioMPs are cited on a level of 6000 times a year. Based on Figure 1, we can say that the field of study of BioMPs lags behind the field of study of MPs by about 5–6 years. In the coming years, this topic will be one of the most popular in modern ecology.

Although this area of research is quite new, there are already some review articles on the impact of BioMPs on the planet. Of these, two are devoted to the impact of BioMPs on the soil and the rhizosphere [15,16]: one to individual materials, i.e., PLA [17], one discussed the risks associated with the formation of BioMPs [18], and another one reports on the interaction with pollutants and influence on organisms [19]. In the last two, the authors state that the main risks, in their opinion, are that BioMPs particles can stay in the environment for a long time and can also be carriers for microorganisms and pollutants. However, to date, no reviews have been found on the impact of BioMPs on some organisms participating in the human food chain: higher plants and aquatic animals (fish and shellfish). This review will focus on this topic.

## 2. Biodegradable Microplastics and Plants

Nowadays, several reviews have analyzed the interactions between nonbiodegradable microplastics and plants, discussing the phytotoxic effects given by MPs uptake [20,21,22]. The most common are plant growth suppression, mechanical damage to the root system, and MPs accumulation in edible parts, as well as the effect on soil properties and its microbiome. However, in response to exposure to microplastics, plants can activate defense mechanisms to reduce the negative impact [23]. Moreover, all these reviews agree that there is currently not much data on the subject and that more field trials are needed.

**Figure 2:**
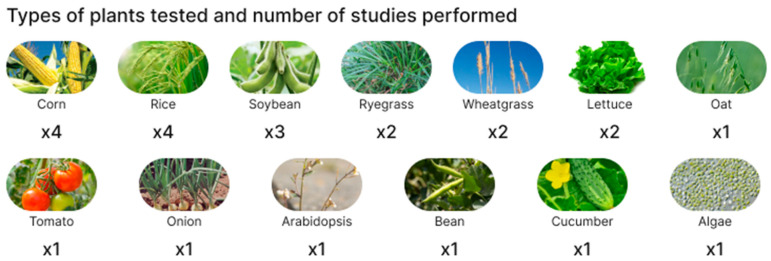
Summary infographics on the type of plants used as models in the reviewed articles.

**Figure 3:**
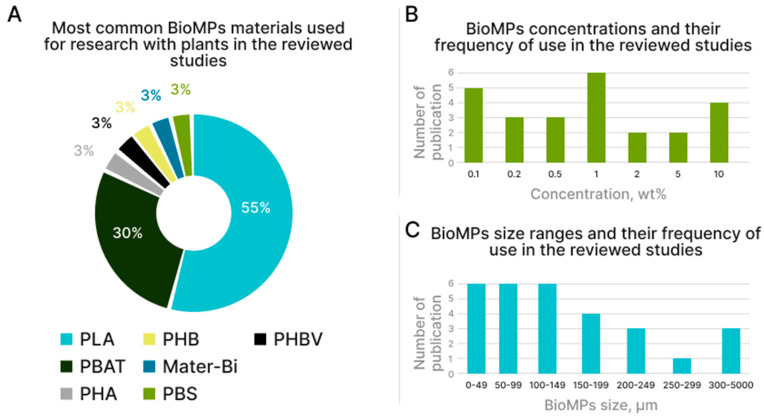
Summary infographics on the characteristics of BioMPs used in the reviewed articles: (**A**) type of materials, (**B**) concentration in soil, and (**C**) average size ranges.

At the time of writing this review (May 2023), 22 research articles have been identified from the Scopus and Web of Science databases investigating the interaction between biodegradable plastic particles and plants [24,25,26,27,28,29,30,31,32,33,34,35,36,37,38,39,40,41,42,43,44,45]. The survey methodology included queries in the Scopus and Web of Science databases for “biodegradable microplastics and plants”. For better detail, the word “plants” was replaced by specific types of “corn”, “soy”, and “rice” from the articles found. Since, at the moment, this field of science is quite young, no time frame was set for the selection of articles. The criterion for inclusion in the review was the availability of results specifically on the effect of BioMPs on the plant itself. The exclusion criteria were as follows: study of nonbiodegradable microplastic particles, lack of an English version of the text. Also, commentaries, summaries, reviews, editorials, and duplicate studies were excluded. Summary infographics on the types of plants tested and on BioMPs characteristics (biopolymer nature, particle size, and concentration in the soil) are shown in Figure 2 and Figure 3, respectively, whereas a summary of the main findings is reported in Table 1.

As can be seen from Figure 2, all tested plants except two (i.e., the Arabidopsis and the Algae) were agricultural crops. In particular, in the 22 reviewed articles, rice and corn appear 4 times each; soybean 3 times; ryegrass, wheatgrass, and lettuce 2 times each; and oat, bean, algae, tomato, onion, and cucumber 1 time each. Therefore, 14 publications were on monocots (corn, rice, ryegrass, oat, wheatgrass, onion, and algae), and 8 on dicots (soybeans, lettuce, bean, tomato, cucumber, and arabidopsis), with a total of 7 monocot and 6 dicot species tested.

For cultivating corn and soybeans, mulch film is used, which is gradually being replaced by a biodegradable [46,47]. Therefore, the study of the effect of BioMPs on the growth of these plants is in demand in the first place. Additionally, these plants are important for the human food chain. Corn is most popular in the regions of Central America and Africa [48]. Soy is one of the staple foods in Asia [49]. Furthermore, soybeans produce a huge amount of feed for livestock and birds due to their high protein content [50]. A change in the yield of these crops can have great consequences for humanity.

The most studied BioMPs were made of PLA and PBAT. These polymers, in fact, are the most common biodegradable substitute for LDPE in the production of mulching film for agriculture [51].

Regarding the size, the BioMPs under study can be classified into four size ranges, from particles smaller than 50 µm to 2 cm, all having a quite uniform size distribution (Figure 3). Since, currently, there is a lack of research to estimate the size of BioMPs particles formed in the soil, we can focus on the typical dimensions of the formed MPs, which, anyway, can differ in different worldwide regions [52,53]. In fact, some authors identified, quantified, and measured the sizes of the most commonly produced MPs in soils of specific regions, finding that, in the southwestern region of China, ca. 82% of MPs are in the size range of 50–250 µm [52], while in Switzerland the highest MPs concentrations are associated with particles of larger size 125–500 µm [53].

Since there are no exact data on the concentration of BioMPs in soils, researchers are mainly guided by data on the content of MPs. Initial quantitative estimates suggest that background concentrations may be as high as ~0.002% of the soil weight in Swiss nature reserves [54]. Levels > 7% by weight of soil have been reported in roadside soils near industrial areas [55]. In the analyzed articles, the most studied concentrations are in the range from 0.1 to 1 wt%. There are also descriptions of high BioMPs concentrations of about 10% (Figure 3). It is also worth noting that all the studies found were conducted in pots, except only one study [39]. This may introduce distortions in the results obtained. Field tests should be the next stage of research.

Thus, it can be noted that the field of studying the interaction of BioMPs with plants is young and very popular. Research is carried out on different plants with different materials and particle sizes. This will help to accumulate the amount of data and understand the general trends in the interaction of BioMPs with plants at different stages of life.

### 2.1. BioMPs Effects on Seed Germination, Root System Development, and Biomass Growth

Several authors investigated how BioMPs may affect seed germination and their early development since this interaction can have drastic effects on agricultural production yields. Serrano-Ruiz et al. examined the effect of biodegradable mulch film microplastic particles (based on PLA, PHB, and PBAT/starch and PLA/starch blends) in two greenhouse-scale pot assays on tomatoes (*L. esculentum*) and lettuce (*L. sativa*) seeds [24]. The authors’ results provide evidence that BioMPs fragments did not affect germination but have the potential to interact with tomato and lettuce plants and alter plant development. The authors emphasize the danger of this since mulch film is usually used already at the stage of sprouts, not seeds.

The effect on the percentage of seed germination and growth is shown by Li and co-workers, who performed a pot experiment under field conditions using a biodegradable mulch film consisting of a PLA–PBAT blend and soybean (*Glycine max*) as a model plant [25]. With an increase in the concentration of microplastics based on PLA–PBAT, seed germination decreased. In the presence of BioMPs at concentrations of 0.1, 0.5, and 1 wt%, the germination rate was 50, 33, and 17%, respectively, whereas, in the control, the germination rate was 70%. The authors associated this with a change in soil properties and a slowdown in the absorption of water by seeds. However, no negative effect on the growth rate was found, and the highest rate was observed in samples with the addition of 1% BioMPs. A decrease in seed germination was also noted by Boots et al. [26] in a study performed on perennial ryegrass (*Lolium perenne*). With the addition of PLA, 6% fewer seeds germinated compared to the control. In the same study, the effect of PLA on shoot length was shown to be reduced by 19%. The authors give assumptions about the reasons for the decline in germination and development. On the one hand, this may be due to the clogging of pores in the seed capsule; on the other hand, soil microbes contribute to the decomposition of PLA into oligomers, which can interact both with seeds and change soil properties. Su and coworkers [27] demonstrated the effect of PLA and PBS BioMPs on the growth of edible algae (*Chlorella vulgaris*). Both types of BioMPs had a negative effect on the growth rate of algae, which depended both on the type of MPs and on the concentration. In the presence of 100 mg/L PLA and PBS, 47 and 36% fewer algae grew compared to the control. A similar degree of inhibition was achieved in the presence of PE at a concentration 10 times higher. The authors attribute this effect to the size of the particles, as well as their ability to biodegrade and the possible release of chemicals from MP particles.

Yu et al. [28] examined the effects of PLA BioMPs derived from biodegradable disposable masks on the germination and growth of winter grazing ryegrass (*Lolium perenne*). The experiments were carried out in pots, mixing the test soil with BioMPs, both in their original state and after artificial aging in different liquid media (alkaline solution at pH = 10, seawater, aquaculture water, and Fenton’s reagents), at a concentration of ca. 1.2–1.3 g/cm^3^. The authors reported that the presence of BioMPs generally reduces the germination rate of ryegrass seeds. With respect to the control, for which 95% of the seeds sprouted, in the presence of not-weathered particles, there was a reduction by 3 ÷ 8%, and, after their weathering in alkali, seawater, aquaculture water, and Fenton’s reagents, the reduction was by 60%, 79%, 82%, and 53%, respectively. The results were attributed to the blocking of the pores in the ryegrass seed capsules by fine-fiber microplastics and bead-like particles.

Note that, despite the small amount of data, the effects of BioMPs on plant germination were always found to be nonpositive: neutral or negative. To date, there is no consensus on the reasons for this decrease in seed germination. The main proposed mechanisms are changes in soil properties, release of toxic products during decomposition, and blocking of seed pores. It can be assumed that large particles mostly affect germination by changing soil properties and releasing toxic degraded matter, whereas the smaller ones (for sizes around 300 microns and lower, as in articles [25,26,27]) can also enter the pores in the seeds blocking them.

**Figure 4:**
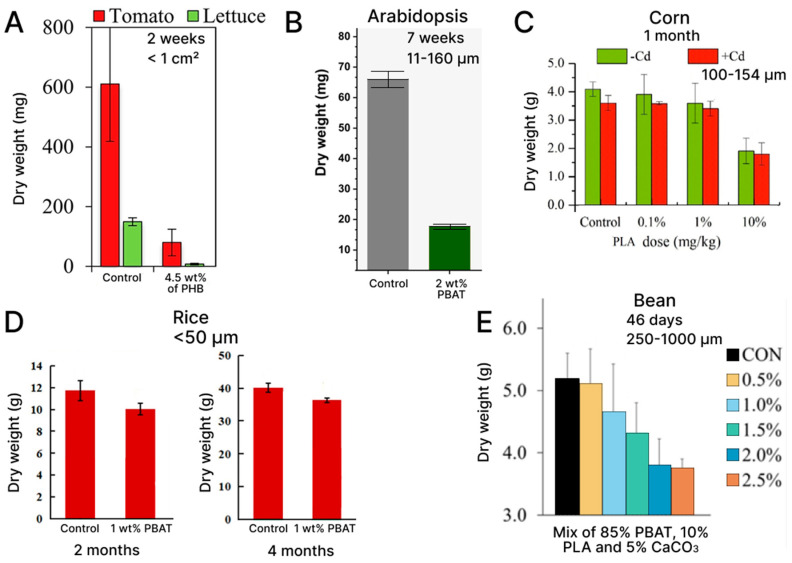
Change of biomass diagrams from different articles: (**A**) Tomato (*L. esculentum*) and Lettuce (*L. sativa*) and 4.5 wt% PHB (size < 1 cm^2^) [24]; (**B**) Arabidopsis (*Arabidopsis thaliana*) and 2 wt% PBAT (size range 11–160 μm) [29]; (**C**) Corn (*Zea mais* L.) and PLA (size range 100–154 μm) [31]; (**D**) Rice (*Oryza sativa* L.) and 1 wt% PBAT (size < 50 μm) [41]; (**E**) Bean (*Phaseolus vulgaris* L.) and PBAT/PLA/CaCO_3_ 85/10/5 (size range 250–1000 μm) [36].

Changing biomass, root system structure, and other plant-growth parameters can affect the yield. Therefore, it is necessary to trace the influence of BioMPs on these parameters. In the article by Liu et al., the mechanisms responsible for the effects of PBAT BioMPs on the biomass growth of Arabidopsis (*Arabidopsis thaliana*) have been investigated. The study demonstrated that the incorporation in the soil of PBAT BioMPs at a concentration of 2 wt% reduced the area of rosette leaves by two times compared to the control and decreased the plant biomass by 27% (Figure 2, Figure 3 and Figure 4) [29]. In addition, the number of pods per plant was about a third of normal, and both the production rate of reactive oxygen species (ROS) were indicative of oxidative stress and the activities of the key enzymes involved in ROS metabolism were significantly increased. The authors suggest that plant-growth suppression is due to the activity of microorganisms that degrade PBAT BioMPs, forming toxic compounds (adipic acid, terephthalic acid, and butanediol). However, this requires further research. In contrast, in another study by Yang et al. on corn seeds (*Zea mays* L. var. Wannuoyihao), PLA increased plant biomass at concentrations of 0.1 and 1 wt%; however, when the dose was increased to 10 wt%, the biomass of wheatgrass decreased to 40%, and the biomass of roots to 50% [30]. The authors present three possible pathways for the effect of BioMPs on plant growth: alteration of soil properties, alteration of soil microbiome and soil enzymes, and release of secondary metabolites during degradation. They suggest that some dose-dependent metabolites may be released during the microbial degradation of PLA, resulting in different effects on plant growth. In another study by the same research group, this also on corn seeds (*Zea mays* L. var. Wannuoyihao), the authors reported that PLA at a 10% concentration strongly inhibited corn growth, reducing the plant biomass and chlorophyll content, but had no significant effect at 0.1% and 1% doses (Figure 4) [31]. Here, the toxicity was attributed to the release of toxic substances during the decomposition of particles, which alter the symbiotic microbial community with possible risks to soil–plant systems.

Different effects of the influence of PLA and PBAT were found in the publication by Cao et al. [32]. At a concentration of 2.5 wt%, PLA reduced the root length of wheatgrass (*Triticum aestivum*) by 39% and PBAT increased it by 72%. However, when the PLA concentration was 0.5%, the root length increased by 55%. Anyway, the changes depend not only on the type and level of biodegradable material but also on the type of plant. In another study, Lian et al. showed that when growing soybean (*Glycine max* L.) the root length was significantly reduced in the presence of 0.1% PLA but increased under 1% PLA, with a dose-dependent effect [33]. A similar alteration was observed in the levels of several antioxidative enzymes involved in ROS scavenging, which indicates the disruption of the antioxidant defense system of the soybean. The authors conclude that different PLA BioMPs concentrations had diverse impacts on the change of metabolites involved in plant growth, reasonably due to an alteration of the soil’s microbial community.

The 0.1–10 wt% concentration range of PLA BioMPs was used by Liu et al. in interaction with corn (*Zea mays* L.) [34]. The article shows that the presence of 0.1% PLA does not affect the biomass of the corn sprout. However, 1% PLA, 5% PLA, and 10% PLA reduced the shoot biomass of corn by 32%, 63%, and 69%, respectively, in comparison with the control. A similar effect of reducing plant biomass in the presence of PLA BioMPs was also reported by Song et al., who used rice (*Oryza sativa* L. var. Yueguang) as a model plant [35]. The authors of both publications attribute this to the degradation of PLA and the release of water-soluble low-molecular-weight oligomers, which induce microbial immobilization and assimilation of essential nutrients and increase stress in plants.

Meng et al. showed that a mixture of PLA and PBAT in an amount of 1.5 to 2.5% reduced the biomass of the roots of beans (*Phaseolus vulgaris* L.), and at concentrations of 2 and 2.5%, the biomass of fruits and the sheet area, also [36]. At all concentrations, higher values of root length were noted, which correlated with the previous results. The authors suggest as possible reasons for this behavior the alteration of the soil microbiome due to BioMPs or the need for plants to increase the length of the roots to search for nutrients at depth.

The reduction in corn (*Zea mays* L.) biomass in the presence of PHBV microplastics was reported by Brown et al. [37]. A significant negative effect was noted already at concentrations equal to 1 wt%. Authors attribute this effect to the rapid influx of labile C substrates into the soil, leading to alleviation in metabolic C limitations.

**Figure 5:**
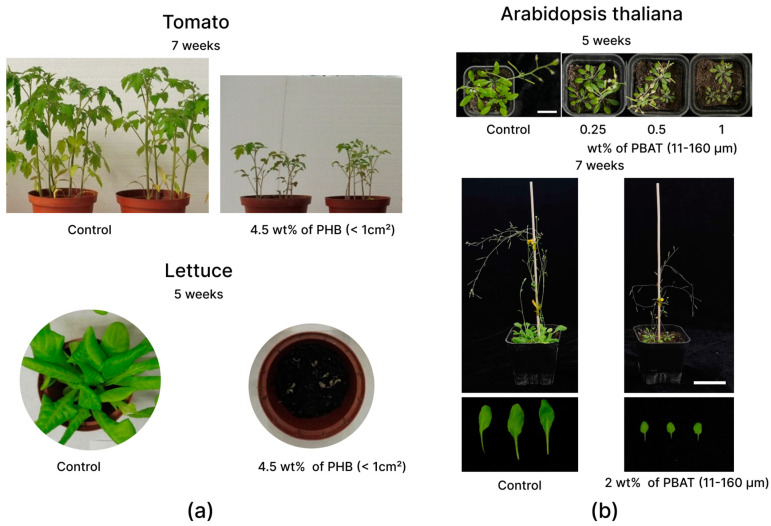
Comparison of the appearance of plants with the control: (**a**) tomato (*L. esculentum*) (**upper rows**) and lettuce (*L. sativa*) (**lower rows**) plants grown for 7 and 5 weeks, respectively, in pots with substrates containing BioMPs PHB (size < 1 cm^2^) [24] (**b**) Arabidopsis (*Arabidopsis thaliana*) after 7 and 5 weeks with BioMPs PBAT (size range 11–160 μm) [29].

In the articles referred to above, pristine BioMPs were used, which is easier from the viewpoint of the experiment, but shows a distorted result. Serrano-Ruiz et al. compared pristine and weathered BioMPs on two horticultural crop plants: tomato and lettuce [24]. The experiment showed that, when using weathered BioMPs based on PBAT + starch and PBAT + PLA, the plant biomass decreased from 37 to 76%, respectively. This effect was noted for both the tomato and lettuce (Figure 5). The authors attribute this effect to the accelerated release of toxic substances from the BioMPs particles. The authors also argue that it is necessary to use weathered particles to study phytotoxicity.

Qi et al. conducted a study about the effects on wheatgrass (*Triticum aestivum*) growth of two different plastic mulch film residues, one made of LDPE and the other one reported as “biodegradable” mulch film [38]. This “biodegradable” film consisted of starch, PET, and PBT in ratios of 37.1%, 44.6%, and 18.3%, respectively. The authors point out that this film cannot be considered fully biodegradable, but this composition is widely used in agriculture. The article showed that microplastics at a concentration of 1 wt% based on this “biodegradable” film showed a stronger negative impact compared to polyethylene. However, the disturbance caused by MPs was offset by the presence of earthworms in the soil. Unfortunately, no other studies on similar effects associated with earthworms and BioMPs were found.

Chu et al. conducted field trials on the effects of two shapes (fiber and powder) of pure PLA on oat (*Avena sativa* L. cv. Bayou 14) and soybean (*Glycine max* L. Merr. cv. Jizhangdou 2) for 5 months [39]. They report that BioMPs at 0.2 wt%, which is indicated as a realistic pollution level of agricultural soils, had no significant effect on soil enzyme activity, soil physicochemical properties (soil moisture content, pH, etc.), root characteristics, plant biomass, and yield. The authors draw encouraging conclusions that PLA BioMPs are not dangerous in the field for a period of up to several months.

Similarly, Souza et al. reported that PBAT BioMPs from mulch films, before and after photodegradation and biodegradation in soil, did not induce phytotoxicity in lettuce (*Lactuca sativa*) and were not cytotoxic and genotoxic for onion (*Allium cepa*) [40]. Anyway, they also stress the need for additional studies to complement the ecotoxicological impact assessment, as also suggested by the European Committee for Standardization—EN, 17033/2018 [56].

Thus, it can be noted that the effect of BioMPs on plants depends on several factors: the type of particles, the concentration, and the type of plant. Large concentrations (>2%) most often had a negative effect on plant and root biomass. Simultaneously, in almost all cases, an increase in the length of the plant root was noted. It can be assumed that this is due to the search for less polluted soil layers in depth or an increase in plant resistance since microplastic particles change soil parameters and make it looser.

It is also worth noting that no correlation was seen between BioMPs particle size and plant biomass. In general, the studied BioMPs particles had a size of up to 150 μm, and, in all cases, they had a negative effect on the plant and root biomass.

### 2.2. BioMPs Effects on Internal Processes of Plants

In addition to influencing external factors, BioMPs cause disturbances in the internal processes of plants. Most often, the presence of BioMPs leads to oxidative stress in plants. Liu et al. performed a study on Arabidopsis (*Arabidopsis thaliana* L. Heynh.) that, although not of any agronomic importance, is one of the most used model organisms for plant sciences [29]. The authors have shown that PBAT particles increased the rate of ROS production in the leaves of Arabidopsis after 14 and 28 days. Also, the presence of PBAT increased the content of malondialdehyde (MDA) after 28 days. Both of these factors indicate oxidation processes.

Also, an increase in the content of MDA and ROS in the presence of PBAT was reported by Yang and Gao [41]. In the shoots and roots of rice (*Oryza sativa* L.), they were induced significantly more than 2 months after sowing. However, the concentration of MDA and ROS was lower than with the addition of PE microplastic particles. The authors suggest that MPs and BioMPs affect plant growth through nitrogen metabolism and photosynthesis.

In addition to increased ROS and MOD concentrations, there have been reports of changes in peroxidase (POD) activity. POD is an antioxidant enzyme that functions in animal and plant physiological defense strategies against free radicals and ROS generated due to biotic and abiotic stresses [57]. Impairments in the work of the POD were reported by Lian et al. [33]. The addition of 0.1% PLA MP decreased POD activity by about 30%. The metabolomics study suggested that the significantly affected metabolic pathway is amino acid metabolism.

There are also data on changes in the process of photosynthesis in plants under the influence of PBAT. Yang and Gao noted the suppression of genes involved in photosynthesis, as well as antenna proteins in rice in the presence of PBAT [41]. In this regard, the rate of net photosynthesis decreased after two months of the experiment; however, this effect disappeared after 4 months of the experiment. This may indicate that with the development of the plant, the negative effects can be leveled.

Changes in the functioning of the corn (*Zea mays* L.) antioxidant system have been reported by Sun et al. [41]. The presence of the BioMPs mixture at concentrations of 1 and 10%, consisting of PBAT and PLA, increased the concentration of H_2_O_2_ in the corn leaves by three times compared to the control. However, similar to Yang and Gao [40], the authors showed that corn sprouts adapted to stress over time and regulated the activity of antioxidant enzymes.

Thus, it can be noted that in all the articles found the presence of BioMPs affected the antioxidant system of various plants. Generally, an increase in the concentration of ROS was noted, which indicates oxidation. There is initial evidence that plants can adapt to this by regulating the activity of enzymes. Also, oxidation may decrease as the plant grows.

### 2.3. Interactions of BioMPs with Heavy Metals and Effects on Plants

In addition to soil contamination with microplastic particles, there is the problem of the accumulation of heavy metals in it. To date, it has been shown that different types of MPs can adsorb heavy metals on their surfaces and facilitate their movement in the environment and into living organisms [58,59,60]. However, there is still no clear understanding of the interaction between MPs, heavy metals, and plants. On the one hand, some studies have shown that MPs can adsorb heavy metals and adhere to the root surface, and then, facilitate their penetration into plant roots through apoplastic or symplastic pathways [32,61]. However, MPs particles attached to the root surface can prevent the absorption of heavy metals by competing with heavy metals for adsorption sites on the root surface. Further, the results of studies on the interaction of BioMPs, heavy metals, and plants will be considered.

Most often, researchers reported on the effect of PLA-based MPs on the accumulation of heavy metals in roots without changing their concentration in shoots. The study by Lin et al. [43], performed on rice (*Oryza sativa* L.) as a model plant, showed that at a PLA concentration of 0.2 wt% and a Cd concentration of 1 mg/kg, Cd accumulated in the roots in an amount about 50% higher compared to the control. The authors hypothesize that the causes are alterations in soil pH and microbial communities due to the biodegradation of BioMPs. However, an opposite result was reported by Wang et. al. on the growth of corn (*Zea mays* L. var. Wannuoyihao) exposed simultaneously to MPs and Cd [31]. In their study, the authors showed that the presence of PLA MPs did not significantly change the Cd concentration in both roots and shoots except at the dose of 10%, to which the Cd uptake by the plant was appreciably reduced. They attributed this result to the decreased plant biomass due to severe phytotoxicity produced by the high-dose PLA MPs. A similar effect was reported by Liu et al., once again on rice [44]. However, the reduction in Cd levels in rice was already significant with 2% PLA microplastic. The difference in the result can be explained by the difference between the plants—rice or corn—under study; it can be assumed that different plants tolerate the interaction with BioMPs and heavy metals in different ways.

Metal accumulation in corn (*Zea mays* L. var. Wannuoyihao) was measured by Yang et al. [30], where the presence of PLA particles increased the Zn content in the roots in the presence of ZnO in the soil. Simultaneously, the Zn concentration in the shoots did not change, and, in some cases, even decreased. In the absence of ZnO in the soil, high PLA concentrations (10%) also reduced the Zn concentration in the shoots while increasing it in the roots. The authors suggest that a big concentration of BioMPs changes soil properties since they may attenuate the soil retention of heavy metals via a “dilution effect”, so ultimately increasing Zn accumulation by roots.

In a study investigating the interaction of BioMPs with antimony of different oxidation states (Sb(III) and Sb(V)) on wheatgrass seed development, it was found that, in addition to the type of material, the oxidation state of antimony affects the penetration into plants [32]. At high concentrations of 2.5%, PLA particles contributed to the accumulation of both Sb(III) and Sb(V) in the wheatgrass roots. Simultaneously, PHA particles at the same concentration did not show any effects when Sb(III) was introduced but significantly increased the Cd concentration in the presence of Sb(V).

Sun et al. used a mixture of PLA and PBAT microplastics to investigate the effects of BioMPs on photosynthesis, antioxidant defense systems, and arsenic accumulation in corn (*Zea mays* L.) seedlings growing in arsenic-contaminated soils [42]. They found that BioMPs are phytotoxic in As-contaminated soils at all considered concentrations (0.1, 1, and 10%), and the effects are higher than those given by polyethylene MPs used for comparison. Moreover, they proved that BioMPs at 10% reduced the leaf area and inhibited the accumulation of As in corn seedlings, maybe due to the inhibition of chlorophyll synthesis and photosynthetic rates in the corn seedlings’ leaves, and to BioMPs’ capacity to bound As in the soil, thereby reducing its bioavailability for the plant. The change in the bioavailability of HMs due to the presence of BioMPs in the soil was also reported in a large study by Li et al. [62].

Zhang et al. studied the effects of different types of MPs, made of biodegradable and nonbiodegradable polymers, on Cr accumulation and toxicity to cucumber (*Cucumis sativus* L.) in hydroponics, keeping cucumber sprouts in various solutions containing MPs or BioMPs and Cr(VI) [45]. MPs, regardless of the type, changed the accumulation of Cr, plant growth, and the defense system of cucumber plants upon treatment with Cr(VI), which was mainly determined by the MP type and particle size. PLA-based BioMPs reduced the accumulation of Cr(VI) due to the high adsorption capacity for Cr(VI) in the solution. This demonstrates that BioMPs particles can inhibit HMs not only in soil but also in water.

Thus, based on a few articles, it can be concluded that BioMPs can affect the accumulation of heavy metals in plants. The main places of accumulation are the roots of the plant. However, the effect depends on a combination of several factors: the type of metal, the type and concentration of the BioMPs, as well as the plant itself. It can be noted that the presence of heavy metals and BioMPs in the soil has a double effect. On the one hand, cases were recorded in which the concentration of HMs in plants decreased. On the other hand, the presence of BioMPs had a negative effect on plants and reduced germination and biomass. Therefore, the area of interaction between heavy metals and BioMPs needs to be explored further to systematize the possible effects and understand the risks. In this area, further research is needed to be able to better systematize the effects obtained.

**Figure 6:**
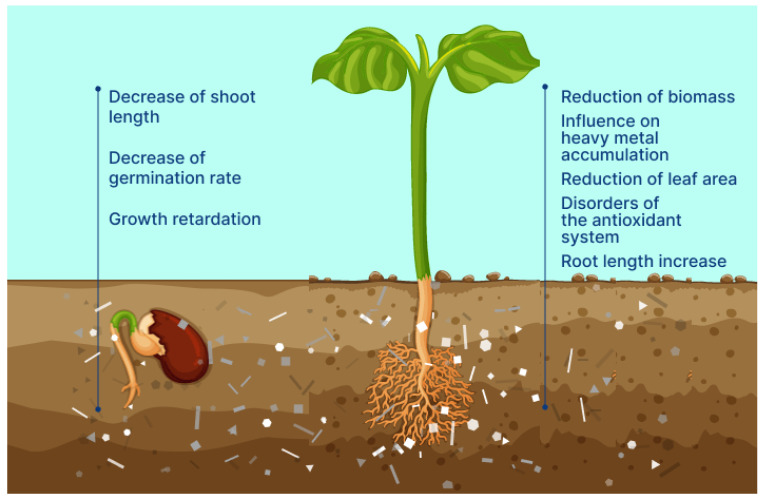
Potential negative influence on plants by biodegradable microplastics.

Possible negative effects in the interaction of BioMPs and plants are presented in Figure 6. At the stage of germination, the presence of BioMPs in the soil can reduce the germination of seeds and cause delays in the development of the plant. In grown plants, BioMPs can adversely affect biomass and the area of leaves and cause violations in the operation of the antioxidant system. Thus, it is necessary to continue research in this area and the development of new standards for the disposal of biodegradable plastics.

## 3. Biodegradable Microplastics and Fish

Another source of food for humans besides soil is water. Many reviews and books have been devoted to the microplastic pollution of the hydrosphere [60,63,64,65,66]. They all agree that microplastic particles adversely affect it. In addition to interacting with aquatic biota, MPs can adsorb heavy metals and bacterial colonies on their surface. The toxicity of MPs in water depends on the type of plastic, concentration, and size while lowering increases their bioavailability and harmfulness [60].

The presence of microplastics in water is dangerous for humans not only by direct ingestion but also by secondary ingestion, which can be channeled through the food web [65]. Therefore, it is necessary to understand how microplastic particles affect animals. To date, there are large reviews on the interaction of nonbiodegradable microplastics with animals [67,68]. These reviews show that microplastic particles adversely affect the gastrointestinal tracts of animals, can accumulate in the body, and affect the reproductive system. The authors of the reviews and studies reviewed in them indicate the need to continue studying this area. The researchers also refer to the need to harmonize approaches in studying the impact of microplastics on animals.

The annual global per capita consumption of seafood is approximately 22 kg [69]. This tells us that seafood has an important place in human nutrition. Therefore, the impact of the possible harmful effect of microplastics, including biodegradable ones, on seafood, and especially fish, can greatly affect food humanity. Review articles on the impact of BioMPs on aquatic life were not found at the time of writing this review; this is because the topic is quite new and not enough data has yet been accumulated. Thus far, research is aimed at studying the mechanisms of BioMPs formation in aquatic environments [70,71]. The infancy of the BioMPs and animal sphere is indicated by the fact that there are no generally accepted research protocols yet. Methods for detecting and isolating these particles from tissues are being developed [72].

The number of articles related to BioMPs and fish is poorly significant, so it is not very appropriate to make statistics on them. The survey methodology included queries in the Scopus and Web of Science databases for “biodegradable microplastics and fish”. For detalization, the word “fish” was replaced by specific types of “zebrafish”, “commercial fish”, “bass”, and “shellfish” from the articles found. The exclusion criteria were the same as with plants and as follows: study of nonbiodegradable microplastic particles and a lack of an English version of the text. Also, commentaries, summaries, reviews, editorials, and duplicate studies were excluded.

The found articles, summarized in Table 2, used BioMPs concentrations from 0.1 to 500 mg/L, but concentrations around 20 mg/L are most used. It is still difficult to assess how relevant this concentration is for BioMPs because the concentration of common MPs in the North Pacific Subtropical Gyre (NPSG) was 250 mg/L in 2012 [73]. Given the increase in plastic pollution, this concentration will increase. Also, from Table 2, it emerges that the most frequent BioMPs’ sizes are up to 350 μm and the most studied material is PLA. Generally, BioMPs are added to the medium for incubating embryos; in the case of adults, they are added to water. However, Xie et al. [74] used a technique to add microplastic particles to each feed pellet, which ensured that they entered the gastrointestinal tract. Based on the foregoing, it is worth noting that, so far in the field of studying the interaction between BioMPs and animals, the situation is similar to MPs; BioMPs studies are conducted without a standard protocol, and different concentrations, sizes, and ways of getting BioMPs into fish organisms are considered.

Zebrafish (*Danio rerio*) are the most used animal models for microplastic toxicity studies. They are a near-perfect model for toxicity studies due to their small size, ease of breeding, short life cycle, and inexpensive maintenance [75]. They also have transparent embryos, which makes them easy to study, and have a genetic similarity to humans (70% of human genes are found in zebrafish), which can help predict the resulting effects.

According to the literature, BioMPs have begun having a negative effect already on zebrafish (*Danio rerio*) embryos. Zhang et al. showed that the presence of BioMPs from PLA does not have a cardiotoxic or lethal effect on embryos but inhibits their skeletal development [76]. Moreover, they found that the photodegradation aggravated the PLA BioMPs’ toxicity due to higher levels of bioaccumulation attributed to the rougher surface that can better cling to the gastrointestinal tract epithelium. Consequently, they observed increased adverse effects on several associated physiological responses, (mitochondrial damage, apoptosis in larvae, skeletal development inhibition, and increased levels of reactive oxygen species that cause damage to lipids, proteins, and DNA). A similar effect was shown by Zhang et al. [77] in a study investigating the adverse effects of photoaged polyamide MPs on the growth, intestinal function, and lipid metabolisms of zebrafish (*Danio rerio*) as the model organism. The authors found that photoaging aggravates the adverse effects of MPs, which bioaccumulate in the intestinal tract more than the pristine ones. This determines higher levels of oxidative stress and higher suppression of lipid digestion and absorption, with consequent inhibition of the larvae zebrafish growth. Therefore, it can be stated that photodegradation makes MPs and BioMPs more dangerous for living organisms and plants. Based on these studies and other current research, the negative effects of environmental aging on MPs’ safety seem a quite general result, as it comes out from recent literature on MPs weathered in different environments [24,78]. This raises certain concerns since it is aged MPs that are most often found in the environment. However, for a better evaluation of the MPs’ toxicity to the ecological environment and human health, more work is required to simulate various aging processes on a long-term timescale and to increase the environmental relevance of laboratory simulation through accelerated weathering tests and systematic investigations aimed at a deep understanding of the role of MPs’ size, morphology, and actual concentrations in the environment.

In another publication, de Oliveira and coworkers examined the effects on the behavior and development of zebrafish (*Danio rerio*) larvae caused by their exposure to unaged PLA BioMPs at two different concentrations (3 and 9 mg/L), chosen as representative of the pollution levels by MPs in highly polluted freshwater ecosystems [79]. They found that the ingestion of PLA BioMPs affected the motor and exploratory activity of larvae, which reduced their swimming distance and speed in an open-field test and induced a long time of immobility, which was related to anxiety-like behavior and interpreted as indicative of a BioMP’s neurotoxic effect. Moreover, the authors also reported the accumulation of BioMPs by larvae and the inhibition of acetylcholinesterase (AChE) activity, a neurotoxicity marker that has a central role in controlling various metabolic, physiological, and behavioral aspects.

When examining adult zebrafish (*Danio rerio*), researchers noted the accumulation of BioMPs particles in their bodies. Chagas et al., in a toxicity study performed in an aquarium using PLA BioMPs at concentrations of 2.5 and 5 mg/L, found that the particles accumulated in the liver, brain, gills, and animal carcasses [80]. However, the authors report that the accumulation did not cause movement disorders or anxiety-like behavior in the adult zebrafish, but they note an increase in AChE activity and an imbalance in oxidation-reduction systems, which weakened the protective reaction of fish against predators. In addition, the authors noted an altered pigmentation pattern in the fish, which may have long-term consequences. Data in the present research suggest that PLA BioMPs have adversely affected the cholinergic nervous system and it had potentially negative consequences on nervous and neuromuscular functions. In more recent work, this was also performed in an aquarium; Duan et al. exposed adult zebrafish (*Danio rerio*) to bio-based PLA BioMPs and conventional petroleum-based PET MPs (at a concentration of 25,000 particle·L^−1^) for 15 d to compare the fish preference for diet and the different toxicological impacts of the two types of particles [81]. First, zebrafish demonstrated a distinct diet preference for PLA BioMPs than for PET MPs, as can be inferred from the number of particles found in the gut; after 5 days of the experiment, the PLA particles were 2568 ± 356 and the PET ones were only 87 ± 12, about 170 times less. The accumulation of BioMPs in the intestines of zebrafish led to structural changes in the epithelium. The length of the villi decreased by 20% and the number of goblet cells decreased by 53% when compared with the control. Also, the presence of PLA in the intestine led to specific changes in the diversity of the intestinal microbiota and contributed to the promotion of species closely related to energy metabolism, cellular processes, and fish diseases. The changes in the microbiota are associated with the depolymerization of PLA in the intestine, which changes pH. Thus, prolonged exposure to PLA BioMPs in zebrafish can lead to more serious changes; so, this issue remains relevant for further study.

A comparison between the potential toxicological effects of biodegradable and conventional microplastics was also performed by Xie et al. in a study performed on a different fish species, the Asian seabass (*Lates calcarifer*), very important for commercial fishing and fish farming and diffused in a wide range of habitats [74]. In the experiments, juvenile seabass (*Lates calcarifer*) were fed for 21 days with either BioMPs, made of a PLA/PBAT blend in the weight ratio 30/70, or MPs made of PE. Then, their responses to the diet were analyzed evaluating the microplastic accumulation in their bodies and the microplastics’ impacts on the fish’s health, testing their effects on oxidative stress, gut microbiota, and proteomic modifications. The authors reported very few differences between the two types of MPs. Both did not lead to lethal outcomes and did not cause a significant antioxidant response after short-term exposure, except for glutathione reductase activity changes. However, the accumulation of BioMPs in the gut maintained higher intestinal microbial diversity than the PE MPs but also induced greater liver proteome alteration due to the suppression of proteins associated with immune homeostasis. Anyway, the authors highlight the need for deeper studies aimed at incorporating the effects of long-term exposure to microplastics and the analysis of individual-level response.

Jang et al. investigated the potential of PLA BioMPs in transport to other environments and transfer to ingesting organisms the pollutants (trace metals such as Cu and Pb, and pathogenic bacteria) that can stick on their surface [82]. For their study, the researchers used as a model organism the African sharptooth catfish (*Clarias gariepinus*), chosen for its large commercial importance. The catfishes were reared by exposing them to feeds with or without trace metals (Cu end Pb) and BioMPs and, after three months, they were analyzed for microplastic accumulation in their tissue and the consequent dysbiosis effects. The tests demonstrated that the BioMPs effectively attract both trace metals and bacteria in natural waters and carry them to fish tissue (gills, muscles, intestines, and livers). In particular, the monthly HMs bioaccumulation factors increased 2–4 times with respect to the control. In addition, the uptake of polluted BioMPs altered the microbiome in catfish intestines in a way that reduced the fish’s immunity, as evidenced by the increased counts of Vibrio sp., an opportunistic pathogenic bacterium responsible for serious threats to aquaculture.

Campani et al. carried out a set of ecotoxicity biotests on some model marine organisms at different levels of the marine trophic chain. In particular, they selected a shellfish, the sea urchin (*Paracentrotus lividus*), and a fish, the sea bass (*Dicentrarchus labrax*) [83]. The organisms were exposed to elutriates of marine sediments inoculated with BioMPs made of Mater-Bi (HF03V1 grade), so there was no BioMPs ingestion in the study, only interaction with Mater-Bi degradation products. The authors reported that the Mater-Bi degradation did not generate and transfer into the elutriates toxic substances that cause embryo toxicity towards sea urchin (*P. lividus*) or alterations in responses of sensitive biomarkers (lipid peroxidation, LPO, and erythrocytic nuclear abnormalities, ENA, assay) for the sea bass (*D. labrax*). The authors underline that they did not find other studies in the literature reporting on fish exposure to elutriates of sediment inoculated with plastics to compare their results. Nevertheless, they suggest the possibility that their ecotoxicological approach may become a standardized investigation scheme to assess ecotoxicity due to bioplastic degradation in marine sediments.

Some other research groups used shellfish as target model organisms for studying the toxicological effects of BioMPs in marine environments, focusing their attention in particular on edible species of mollusks [84,85,86,87].

Green analyzed the impact of PLA BioMPs and HDPE MPs on the health and biological activities of European flat oysters (*Ostrea edulis*), and the cascading effects on macrofauna within their benthic habitats [81]. She performed a two-month study in outdoor conditions on a mesocosm scale using intact sediment cores, with the aim of combining a realistic experimental design with high control of the experimental conditions. Two different dose levels of microparticles have been considered: low (0.8 μg L^−1^) and high (80 μg L^−1^). In both cases, no significant alteration of the filtration and growth rates of oysters (*O. edulis*) was detected. However, PLA BioMPs at a high dose modified the respiratory response of the organisms, determining an increase in their respiration rates, which can be interpreted as a symptom of stress. Concerning the impact of microplastics on the associated macrofauna, Green found that both types of MPs at the high dose reduced the number of juvenile periwinkles (*Littorina* sp.) and isopods (*Idotea balthica*) by about two and eight times, respectively. In a subsequent study, also in outdoor mesocosm conditions but having blue mussels (*Mytilus edulis*) as the target model organism, Green et al. showed that PLA BioMPs exposure reduced the health of the mussels since they lowered their attachment strength and changed the immunological profiles of their hemolymph proteome [82]. However, the effects of PLA BioMPs were less severe than those of HDPE MPs. Another mollusks-related study investigated the effects of exposure to PHB BioMPs or PE MPs, alone or in combination with the polycyclic aromatic hydrocarbon fluoranthene (Flu), on blue mussel (*Mytilus edulis*) oxidative stress biomarkers [86]. The authors found that both types of MPs, in single or combined exposure with Flu, exerted metabolic stress since they modified the antioxidant responses of detoxifying enzymes in digestive glands and gills. The alterations were similar for BioMPs and MPs, and no significant combined effects produced by Flu coexposure were noted. Finally, the study by Joyce and Falkenberg [87], which investigated the impact of nonbiodegradable (PET) and biodegradable (PLA) MPs at two concentrations (100 μg/L and 1000 μg/L) for 4 weeks of exposure on the Asian green mussel *Perna viridis*, highlighted no significant effects on the mortality or clearance and the oxygen consumption rate of the organisms. Therefore, the authors concluded that both types of MPs cause only minimal direct effects on bivalve functioning, at least in their model ecosystem.

**Figure 7:**
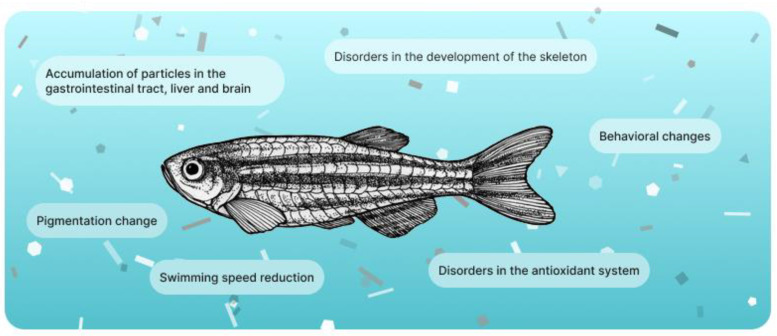
Potential negative influence on fish by biodegradable microplastics.

Thus, from this analysis, it can be noted that, at the moment, the most studied material in the field of interaction with marine animals is PLA. Unlike plants, no articles were found using BioMPs based on PBAT and only mixed with PLA. Based on data obtained using zebrafish (*Danio rerio*), potential risks can be assessed. The presence of BioMPs had no lethal effect on fish embryos. However, bioaccumulation, behavioral, and color changes are noted. In older fish individuals, bioaccumulation of particles, changes in the microbiota, and morphology of the gastrointestinal epithelium are also measured. The presence of BioMPs impacts the antioxidant system of fish (Figure 7), similar to what has already been discussed for plants. In the study of commercial fish, no special ecotoxic effects were observed. It can be assumed that larger organisms cope better with BioMPs biodegradation. Concerning the possible particle size effects on the potential toxicity of BioMPs for aquatic organisms, the literature data do not show a clear trend and are too low to have a critical discussion. Based on the foregoing, it can be emphasized once again that the scope of studying the effect of BioMPs on animals is at an early stage. Researchers should take advantage of the experience gained in the study of MPs. The creation of BioMPs research protocols and the accumulation of data arrays obtained using different conditions, materials, and organisms will allow for the introduction of new standards for biodegradable materials in the industry, which will include not only industrial processing and composting but also the biodegradation of materials in real conditions.

**Table 2:** Studies on the impacts of BioMPs on fish.

Reference	Material	Shape of BioMPs	Size of BioMPs	Concentration of BioMPs	Type of Animal	Time of Exposure	Main Results
[74]	Bio (30%PLA and 70% PBAT)	Cut MPs	ca. 3.06 mm × 2.71 mm	Microplastics are in every piece of food	Asian seabass (*Lates calcarifer*)	21 days	(−) Bioaccumulation in gastrointestinal tracts (−) Induced proteome modulation by downregulating proteins associated with immune homeostasis. (+) Biomicroplastics maintained higher intestinal microbial diversity and induced more protein alteration than PE microplastics.
[76]	PLA and UV-treated PLA	Irregular fragments	5–50 μm	0.1, 1, 10, 25 mg/L	Embryos of Zebrafish (*Danio rerio*)	90 days	(+) PLA exhibited no cardiotoxic or lethal effects on larvae. (−) Photolytic degradation elevated the skeletal development inhibition of PLA on larvae. (−) Bioaccumulation and skeletal development inhibition of zebrafish. (−) More severe mitochondrial membrane depolarization induced by degraded BioMPs. (−) Oxidative stress-triggered mitochondrial structural damage, depolarization, and fission inhibition. (−) Bioaccumulation of PLA MPs in larvae, leading to aggravated toxicity to developing zebrafish.
[79]	PLA	Ground MPs	2.34 ± 0.07 μm	3, 9 mg/L	Zebrafish larvae (*Danio rerio*)	5 days	(−) Decreasing swimming distance and speed (−) Inhabitation of acetylcholinesterase activity
[80]	PLA	Ground MPs	2.34 ± 0.07 μm	2.5 mg/L 5 mg/L	Zebrafish (*Danio rerio*)	30 days	(−) PLA accumulated in the liver, brain, gills, and carcass of the assessed animals. (−) Behavioral changes (in shoal) predictive of cospecific social interaction and antipredatory defensive (−) Changes in the animal’s pigmentation pattern.
[81]	PLA, PET	Irregular-shaped MPs	135.35 ± 37.12 μm 20% of particles were <50 μm, 49% were 50–100 μm, and 31% were >100 μm	17.5 mg/L	Zebrafish (*Danio rerio*)	5 days	(−) PLA BioMPs accumulate significantly more than PET MPs in the zebrafish intestines (−) Gastrointestinal damage in zebrafish. (−) Specific changes in the diversity of intestinal microbiota and promoted species closely linked with energy metabolism, cellular processes
[82]	PLA	n.d.	-	Added to feed at 10 wt%	African sharptooth catfish (*Clarias gariepinus*)	90 days	(−) PLA transferred higher amounts of metals to catfish than expected and also led to increased Vibrio counts in the intestines
[83]	Mater-Bi™	Cut MPs	40 mm × 40 mm	n.d.	European seabass (*Dicentrarchus labrax*)	14 days	(+) Absence of toxic effects detected
[84]	PLA	n.d.	0.6–363 μm	0.8 mg/L and 80 mg/L	European flat oysters (*Ostrea edulis*)	60 days	(−) Increasing the respiration rate of oysters
[85]	PLA	n.d.	0.5–330 μm	25 mg/L	Blue mussels (*Mytilus edulis*)	52 days	(−) Altering the hemolymph proteome
[86]	PHB	n.d.	10–90μm	100 μg/L	Blue mussel (*Mytilus edulis*)	4 days	(−) Modification baseline levels of biomarkers related to oxidative stress in Mytilus edulis.
[87]	PLA, PET	silver glitter particles of hexagonal shape	200 μm diameter	100 μg/L and 1000 μg/L	Asian green mussels (*Perna viridis*)	28 days	(+) Absence of toxic effects detected

n.d.—not defined. “(+)”—positive effect. “(–)”—negative effect.

## 4. Conclusions and Future Remarks

This review has considered the impact of biodegradable microplastics on higher plants and fish relevant to the food chain. Negative changes in these areas can lead to food problems for a large number of people. Since biodegradable plastics can be biodeteriorated by microorganisms into environmentally friendly simple substances, they appear as a strategic solution to counteract the environmental pollution generated by plastic waste. However, when BPs enter a natural environment, it does not provide the proper conditions for their complete transformation but rather, since BPs decompose easily, it favors their fragmentation. This leads to pollution by BioMPs even more serious than that resulting from conventional MPs. Even if the current literature indicates that BioMPs exert ecological impacts roughly comparable to those of nondegradable MPs, it also demonstrates that BioMPs might show more severe toxicity effects under certain conditions, since during degradation they can release higher levels of chemicals (additives, BioMPs-bound pollutants, and degradation intermediates) possibly noxious for the biotic and abiotic components of soils and aquatic environments. Anyway, the study of the interaction between BioMPs and living organisms is at the very beginning, so only a preliminary risk assessment is possible so far.

Interacting with plants in soil ecosystems, BioMPs reduce germination, biomass, and leaf area, affect the accumulation of heavy metals, and lead to disturbances in the functioning of the antioxidant system. There are cases in which plants adapt to the influence of BioMPs by changing the activity of enzymes. Of course, all negative effects depend in a complex way on the type of materials, their concentration, and size. However, to establish any patterns associated with these parameters, it is necessary to increase the number of studies.

In the area of interaction between fish and BioMPs, there are even fewer articles than plants. However, the first conclusions were not encouraging. In tests with zebrafish, it was shown that the presence of BioMPs can lead to disturbances in the development of the skeleton, changes in the epithelium of the gastrointestinal tract, bioaccumulation of particles in the body of fish, and disturbances in the antioxidant system, as well as changes in behavior. A couple of papers with studies on microplastics and large game fish that showed no negative effects give hope that there is a correlation with the size of the organisms.

All of the reviewed articles have some shortcomings that still limit the knowledge about the biophysical effects of BioMPs on living organisms and their habitats.

1. The **actual concentrations of microplastics** in the soil and water are not exactly known. For different regions, they differ depending on geographical, meteorological, and anthropogenic factors. The effective concentrations of BioMPs are also poorly understood. This is due to the low prevalence of biodegradable plastics. Therefore, current studies use approximate concentrations or a wide range of concentrations, up to extremely high ones. An accurate quantification of the MP and BioMPs levels in the environment is required to be able to accurately simulate the real pollution status in future research;
2. The literature findings currently available clearly show that the toxicological effects of BioMPs on living organisms and ecosystems depend in complex ways on many factors, including their composition, concentration, and size. Unfortunately, the relative contribution of each factor to the potential risks associated with BioMPs cannot be easily deduced, due to the lack of comparative lab studies sensitive to the variation of these relevant parameters. One of the most critical neglected points concerns the effects of the BioMPs’ size and shape (i.e., the surface-to-volume ratio, which strongly influences the BioMPs’ degradation kinetics) on their ecotoxicity. Additional analyses are required to bridge this gap in knowledge;
3. Another limitation is the use of **pristine plastics**, mostly under highly controlled conditions. When released into the environment, bioplastics are exposed to UV, moisture, and other chemical–physical and biological factors. This changes their size, structure, surface chemistry, and morphology. According to preliminary data, such BioMPs are more dangerous. Therefore, in future studies, it is necessary to focus on the weathered particles of MPs and BioMPs;
4. Most often, the impact of **only one material** at **a time** is studied; mixtures are rarely used, and their interactions are not considered. Therefore, in future studies, it is necessary to use mixtures of BioMPs and MPs with concentration ratios that correspond to the real situation. With such an interaction, the negative effects of exposure to heterogeneous BioMPs–MPs mixtures can both be leveled and intensified;
5. The next limitation follows from the previous one; in the soil, in addition to the presence of other MPs, **heavy metals** are also present. Currently, there is a small number of studies on the interaction of BioMPs with heavy metals. Based on current studies, it is difficult to draw conclusions about the results of this interaction; in some cases, BioMPs particles prevent the accumulation of HMs in plants. In others, on the contrary, they contribute. Future research should address more complex mixtures of MPs, BioMPs, and HMs;
6. A final limitation of existing research is that the interaction between BioMPs and living organisms, such as higher plants and aquatic animals, are **studied in pots and aquariums**. They are limited systems where all the processes occurring in natural terrestrial and aquatic environments cannot be fully simulated. Therefore, in the future, it will be necessary to move on research to small real ecosystems (e.g., fields and ponds), to provide a more integrated view of microplastic pollution in the complex natural environment.

Further research in this area should be carried out taking into account these aspects. More organisms and plants should be studied over longer periods. Only by building up a knowledge base in this area will it be possible to understand whether we are on the right path to dealing with microplastic pollution. Of course, nature will eventually adapt to exist with microplastic pollution, even inducing new living habitats, but this will still produce damage to those now existing.

## Figures and Tables

**Table 1:** Summary of reviewed studies on the impacts of BioMPs on plants.

Reference	Material	Shape of BioMPs	Size of BioMPs	Concentration of BioMPs	Heavy Metals	Plants	Time of Exposure	Main Results
[24]	PBAT; PHB (Pristine and field weathered)	n.d.	1 cm^2^	4.5 wt%	No	Red Cherry Tomato (*L. esculentum cv*.) and Trocadero Ribera lettuce (*L. sativa cv.*)	5 and 7 weeks	(+) Germination of both plant species was unaffected by any of the BioMPs; (−) PHB (polyhydroxybutyrate)-based BioMPs fragments severely inhibited tomato and lettuce plant growth, by 90% and 95%, respectively. (−) Overall, field-weathered BioMPs caused stronger effects on plants than the pristine unused ones.
[25]	PBAT and PE	n.d.	Three types: 2 cm × 2 cm, 1 cm × 1 cm and 0.5 cm × 0.5 cm	0.1%, 0.5% and 1 wt%	No	Soybean (*Glycine max*)	4 months	(−) Germination viability was significantly inhibited. (−) The inhibitory effect of PBAT BioMPs increased with concentration. (−) Compared to PE MPs, the BioMPs showed stronger positive effects on soybean height and weaker negative effects on leaf area, culm diameter, and biomass. (−) The interaction between plastic types and concentrations had a significant effect on the plant height and leaf area, but no significant effect on the culm diameter and other biomass indices.
[26]	PLA	Microparticles and fibers	0.6–363 μm (MPs) and 2–7 mm (fibers)	0.1 wt%	No	Perennial ryegrass (*Lolium perenne*)	10 days	(−) Exposure to PLA fibers or microparticles inhibited seed germination and reduced shoot height.
[27]	PLA PBS	n.d.	mPLA: 57.41 μm; mPBS: 53.33 μm	10 mg/L, 100 mg/L, 1000 mg/L	No	Algae (*Chlorella vulgaris*)	11 days	(−) The maximum inhibition ratios of the two types of MPs on *C. vulgaris* were 47.95% (mPLA, 100 mg/L) and 34.25% (mPBS, 100 mg/L), respectively (−) Among them, mPLA showed the strongest inhibitory effect on the growth of *C. vulgaris*. (−) The stress caused by MPs can promote the accumulation of photosynthetic pigments and trigger the evolution of antioxidative substances like SOD. (−) The coexistence of MPs and microalgae can aggravate the aging of MPs in terms of surface roughness, reduced strength of some functional groups, charge changes, and toxic chemicals leaching.
[28]	PLA from biodegradable masks	n.d.	n.d.	1.2–1.3 g/cm^3^	Different treatment of masks: weakly alkaline solutions with pH = 10 seawater actual aquaculture water and Fenton reagents	Ryegrass (*Lolium perenne*)	14 days	(−) Decreasing germination rate of ryegrass seeds in the planting soil with added fibrous MPs treated under different conditions.
[29]	PBAT	Powder	11–161 μm	2 wt%	No	Thale cress (*Arabidopsis thaliana*)	7 weeks	(−) PBAT-MPs may be degraded by microorganisms to produce chemicals that are highly toxic to plants (adipic acid, terephthalic acid, and butanediol)
[30]	PLA	n.d.	100–154 μm	0.1, 1, 10 wt%	Yes, ZnO	Corn (*Zea mays* L.)	1 month	(+) Low-dose PLA promoted plant growth (−) High-dose PLA significantly decreased corn shoot (by 16–40%) and root biomass (by 28–50%)
[31]	PLA, PE	n.d.	100–154 μm	0.1, 1, 10 wt%	Yes, Cd (0 and 5 mg Cd/kg soil)	Corn (*Zea mays* L.)	1 month	(−) 10% PLA significantly decreased plant biomass and chlorophyll content. (−) The coexistence of MPs caused no alterations in Cd concentrations in plant tissues, but substantially increased DTPA-extractable Cd contents in soil. (−) Compared to PE, the high dose of PLA produced more significant impacts on Cd bioavailability, plant growth, and AMF community, indicating a possible risk to soil–plant systems.
[32]	PHA, PBAT, PLA	Powder	25–45 μm	0.5–2.5 wt%	Sb(III)/Sb(V) 0.75 mg	Wheatgrass (*Triticum aestivum*)	90 days	(−) PBAT MPs might lead to higher risks to food chains in Sb-contaminated wetland soil than PLA and PHA MPs. (−) Wheatgrass traits were more sensitive to coexposure of Sb (III) and 2.5%PLA/PHA MPs. (−) Coexposure of Sb (III) with 2.5%PLA MPs showed a lower CISD and a higher AWCD and presented stimulation effects on microbial metabolism activities on carbon sources.
[33]	PLA	arbitrarily shaped particles	20–60 μm	0.1 and 1 wt%	No	Soybean (*Glycine max*)	49 days	(−) 0.1% PLA BioMPs significantly decreased the root length by 27.53% when compared with the control. (−) The metabolomic profile of plant leaves was significantly changed by different MP exposure, a dose-dependent effect can be observed in PLA BioMPs treatments.
[34]	PLA	n.d.	90 μm	0.5, 1, 5, 10 wt%	No	Corn (*Zea mays* L.)	30 days	(−) Compared with the control, 1% PLA, 5% PLA, and 10% PLA reduced the shoot biomass of corn by 32%, 63%, and 69%, respectively. (−) Total chlorophyll and carotenoid contents of the corn decreased significantly with increasing levels of PLA BioMPs
[35]	PLA	n.d.	155–180 μm	10 wt%	No	Rice (*Oryza sativa* L.)	30 days	(−) BioMPs significantly inhibited rice growth, possibly by affecting nutrition (−) Diversity of microbial biomass declined in PLA-amended soils, possibly because PLA particles acting as carbon input inhabited the population of bacteria.
[36]	85% PBAT, 10% PLA and 5% calcium carbonate	Ground	250–500 μm (60% of total MPs weight) and 500–1000 μm (40% of total MPs weight)	0.5, 1.0, 1.5, 2.0, 2.5 wt%	No	Bean (*Phaseolus vulgaris* L.)	12 weeks	(−) BioMPs, especially at 1.5%, 2.0%, and 2.5% significantly inhibited the root and shoot biomass. (−) BioMPs produced higher specific root lengths and specific root nodules
[37]	PHBV	n.d.	1–15 μm	0.01, 0.1, 1, 10%	-	Corn (*Zea mays* L.)	8 weeks	(−) PHBV BioMPs loading rates at 1% and above (representing hotspots, or bioplastic accumulation) caused significant changes in the soil metabolome and microbial community, likely associated with changing function.
[38]	Biodegradable mulch film consisting 37.1% Pullulan, 44.6% PET and 18.3% PBT	Cut particles	2.5% of 1 mm to 500 μm, 62.5% of 500 μm to 250 μm and 25% of 250 μm to 50 μm	1 wt%	No	Wheat (*Triticum aestivum*)	4 months	(−) BioMPs significantly reduced the total plant biomass, fruit biomass, and root–shoot ratio
[39]	PLA	Powder and fibers	Powder < 150 μm Fibers < 5 mm and diameter ~90 μm	~0.2 wt%	No	Oat (*Avena sativa* L.) and soybean (*Glycine max*)	5 months	(+) PLA had no significant effect on soil biochemical properties, root characteristics, plant biomass, and ecosystem multifunctionality
[40]	PBAT	n.d.	n.d.	0.2 wt%	No	Lactuca sativa (*Allium cepa*)	5 days	(+) No negative impact
[41]	PBAT	Powder	<50 μm	1 wt%	No	Rice (*Oryza sativa* L.)	4 months	(−) The study suggests that biomicroplastics affect the growth of rice plants via nitrogen metabolism and photosynthesis.
[42]	PBAT and PLA were mixed in a 6.5:3.5 ratio and used as BPMPs material.	n.d.	n.d.	0.1, 1, 10 wt%	As (V) concentration of 40 mg	Corn (*Zea mays* L.)	30 days	(−) BioMPs showed phytotoxicity at 0.1%, 1%, and 10% concentrations in As-contaminated soils. (−) BioMPs in As-contaminated soils have reduced the leaf area and inhibited the accumulation of As in corn seedlings. (+) Corn seedlings adapt to stress by regulating antioxidant enzyme activity.
[43]	PLA	n.d.	200 ± 20 μm	0.2 wt%	Cd 1 mg/kg	Rice (*Oryza sativa* L.)	60 days	(−) PLA increased the Cd concentrations in rice root
[44]	PLA	n.d.	n.d.	0.2, 2%	0 and 5 mg Cd/kg soil	Rice (*Oryza sativa* L.)	3 months	(−) 2% PLA caused the most distinct changes, e.g., the maximum soil pH and root Fe and Mn contents, the minimum plant biomass and Cd accumulation, and the lowest AMF diversity (Shannon index).
[45]	PLA	n.d.	13 μm; 48 μm; 500 μm	0, 40, 200, and 1000 mg/L	Cr(VI) 0, 20, 50, 100, 200, and 500 μmol/L in water	Cucumber (*Cucumis sativus* L.)	14 days	(+) PLA BioMPs reduced the Cr accumulation in plants due to the high adsorption capacity to Cr (VI) in the solution. (−) PLA BioMPs inhibited the photosynthesis of seedlings and caused lipid peroxidation (−) PLA BioMPs slightly enhanced the activities of SOD, POD, and CAT.

n.d.—not defined. “(+)”—positive effect. “(−)”—negative effect.

## Data Availability

The data presented in this study are available on request from the corresponding author.

## Abbreviations

AChE	Acetylcholinesterase
BioMPs	Biodegradable microplastics
Flu	Fluoranthene
HMs	Heavy Metals
LDPE	Low-density polyethylene
MDA	Malondialdehyde
MPs	Microplastics
PBAT	Polybutylene adipate terephthalate
PBS	Polybutylene succinate
PE	Polyethylene
PET	Polyethylene terephthalate
PHB	Polyhydroxybutyrate
PHBV	Poly(3-hydroxybutyrate-co-3-hydroxyvalerate)
PLA	Polylactic acid
POD	Peroxidase
ROS	Reactive oxygen species
